# Beliefs about benefits and harms of medications and supplements for brain health

**DOI:** 10.1016/j.pmedr.2020.101060

**Published:** 2020-01-25

**Authors:** Zachary A. Marcum, Sarah D. Hohl, Douglas Barthold, Oleg Zaslavsky, Eric B. Larson, Shelly L. Gray

**Affiliations:** a School of Pharmacy, University of Washington, Seattle, WA, USA; b School of Public Health, University of Washington, Seattle, WA, USA; c School of Nursing, University of Washington, Seattle, WA, USA; d Kaiser Permanente Washington Health Research Institute, Seattle, WA, USA

**Keywords:** Dementia, Alzheimer’s disease, Supplements

## Abstract

The role of medications and supplements for brain health is a fast-changing and growing field, making it difficult for patients to receive updated and accurate information. The objective of this study was to assess patients’ beliefs about the helpfulness or harmfulness of various medications and supplements on brain health. A convenience sample of adults from an integrated healthcare system completed a web-based survey. Descriptive statistics were used for this hypothesis-generating study. A total of 1661 respondents completed the survey. The majority of respondents were female (77%), between the ages of 51–70 (64%), and white (89%). Across the selected medications and supplements purported to improve a person’s brain health (vitamin E, ginkgo biloba, hormones such as estrogen or testosterone, fish oil, and statins), 46–64% of respondents reported not knowing or skipped the item regarding their helpfulness to improve brain health. One out of four respondents reported benefits of vitamin E and nearly half reported benefits of fish oil on brain health; neither benefit is supported by current evidence. For the two medication classes evaluated for increasing dementia risk (proton pump inhibitors and anticholinergics used as sleep aids), 63–77% of respondents reported not knowing or skipped the item regarding their harmfulness to brain health. Survey respondents largely reported not knowing the potential benefits and harms of different medications and supplements for brain health. Improved health communication on pharmaceutical effects on dementia risk is greatly needed, and its development and dissemination should involve healthcare providers, patients, and media outlets.

## Highlights

• Respondents were unaware of benefits/harms of pharmaceuticals for brain health.
• Fish oil and vitamin E were most commonly thought to improve brain health.
• Nearly one-third thought over-the-counter sleep aids were harmful to brain health.

## 1 Introduction

In the absence of a cure or disease-modifying treatment for late-life dementia, much of the current focus is on prevention (Larson, 2018). One common approach to dementia prevention is taking medications or supplements that may improve brain health and avoiding harmful medications and supplements (Larson, 2018). However, two recent evidence-based reviews of findings from 89 randomized controlled trials reported that evidence is insufficient to support the use of certain medications and supplements for cognitive protection in persons with normal cognition or mild cognitive impairment (Butler et al., 2018, Fink et al., 2018). This evidence was used to inform the 2017 National Academies’ landmark report, “*Preventing Cognitive Decline and Dementia: A Way Forward*” (Downey et al., 2017). Despite this lack of evidence, there is a growing industry of supplements and medical foods claiming to promote brain health (Hellmuth et al., 2019), with approximately 63% of older adults using over-the-counter (OTC) supplements (Qato et al., 2016).

Moreover, evidence is mixed regarding the possibility that certain medications increase risk of cognitive decline or dementia. Two of the most commonly studied medication classes in recent years on this topic are proton pump inhibitors (PPIs) and anticholinergics. Observational studies have found increased, neutral, and decreased risk of dementia among patients using PPIs (Goldstein et al., 2017, Gomm et al., 2016, Gray et al., 2018). In addition, observational studies have reported increased risk of dementia when examining anticholinergic exposure from all medications; however, emerging evidence suggests that risk might be driven by certain anticholinergic sub-classes (Coupland et al., 2019, Gray et al., 2015, Richardson et al., 2018). Despite their widespread use among older adults, it is unknown what patients believe about the risks of these medications for dementia.

Taken together, the topic of pharmaceutical effects on dementia risk – whether for purported beneficial effects or potentially harmful effects on cognition – is a fast-changing and growing field of interest, making it difficult for patients to receive the most updated and accurate information. Thus, our objective was to assess patients’ beliefs about the helpfulness or harmfulness of various medications and supplements on brain health, using a web-based survey of a convenience sample in an integrated healthcare system. This study aims to identify potential discrepancies between contemporary medical research and patient beliefs as a way to target future communication efforts on dementia prevention.

## 2 Methods

### 2.1 Survey design

We created a 28-item survey to address three domains of beliefs about dementia prevention: 1) brain health and general strategies for dementia prevention; 2) antihypertensive use as a potential dementia prevention strategy; and 3) medications and supplements as protective or risk factors for dementia. The focus of the current analysis is on the third domain, which included nine survey items that assessed patient beliefs about medications as protective or risk factors for dementia. Results from the other survey domains have been reported previously (Marcum et al., 2019a, Marcum et al., 2019b). The survey was designed to be administered to members of Kaiser Permanente Washington (KPW), an integrated healthcare delivery system in Washington State. We sought feedback from the KPW Senior Caucus, whose members serve as advocates of healthy aging and advisors to the KPW medical and research community. The study team incorporated this feedback into the final survey instrument; changes between surveys were minimal and included turning open-ended responses into categorical responses and deletion of low priority items.

The survey items in the third domain included questions about a set of specific prescriptions medications and supplements (i.e., vitamins and herbals) that have been commonly studied for their potential benefit on brain health, or have been linked to increased dementia risk. First, two survey items asked whether the respondent was currently taking any prescribed medication to improve brain health and/or any supplements or OTC medications to improve brain health. Respondents were then asked to rate the helpfulness of the following five prescription medications and supplements for improving a person’s brain health: hormones (such as estrogen or testosterone), statins, vitamin E, ginkgo biloba, and fish oil. Response options were: very useful, somewhat useful, not at all useful, or don’t know. Finally, respondents were asked to rate the harmfulness of the following two medication classes: 1) PPIs and 2) OTC sleep aids, which represent a sub-class of anticholinergics commonly used among older adults. Response options were: very harmful, somewhat harmful, not at all harmful, don’t know. We also collected demographic information including age, level of education, gender identity, employment, household income, and race/ethnicity. Respondents could skip any survey item. Of note, only 37 and 32 respondents skipped the questions on the helpfulness or harmfulness of medications and supplements for brain health, respectively, so we combined the responses of not knowing and skipped. The final survey is included in Supplementary Material (Supplementary Figure 1).

### 2.2 Participants

In January 2018, the study team recruited participants through Northwest Health News, a monthly electronic newsletter emailed to KPW members. Information in the newsletter introduced the study and provided a link to the web-based survey, which was hosted on Survey Monkey (www.surveymonkey.com). Participants were eligible if they were aged 18 or older, were KPW members, and could read and write in English. No incentive was offered.

### 2.3 Data analysis

We exported data from Survey Monkey into Microsoft Excel to calculate descriptive statistics for this hypothesis-generating study. On post-hoc analysis, we conducted a stratified descriptive analysis by sex and age categories of the two items on the helpfulness or harmfulness of the various medications and supplements. The Institutional Review Board at the KPW Health Research Institute approved all study materials and procedures.

## 3 Results

### 3.1 Respondents

A total of 1,661 respondents completed the survey. Table 1 describes respondent characteristics. The majority of respondents were female (77%), between the ages of 51–70 (64%), and white (89%). Over half (67%) had obtained a bachelor’s degree and 46% were retired. A total of 5.3% (n = 88) of respondents reported current use of any prescribed medication to improve their brain health, whereas 16.6% (n = 276) reported current use of any supplement or OTC medication to improve their brain health.

**Table 1:** Survey Respondent Characteristics (n = 1661).

	Respondents, N (%)
**Sex**	
Female	1274 (76.7)
Male	317 (19.1)
Non-binary	3 (0.2)
Prefer not to answer	14 (0.8)
Skipped	53 (3.2)
**Age, years**	
18–50	243 (14.6)
51–70	1055 (63.5)
71–80	300 (18.1)
≥81	59 (3.6)
Skipped	3 (0.2)
**Race**	
White	1470 (88.5)
Asian	39 (2.3)
American Indian or Alaska Native	21 (1.3)
Black or African American	18 (1.2)
Other	43 (2.6)
Prefer not to answer	58 (3.5)
Skipped	51 (3.1)
**Ethnicity**	
Non-Hispanic ethnicity	1512 (91.0)
Hispanic ethnicity	42 (2.5)
Don’t know/prefer not to answer	54 (3.3)
Skipped	53 (3.2)
**Education**	
Bachelor’s degree or higher	1120 (67.4)
Some post-secondary education	407 (24.5)
High school graduate or GED	72 (4.3)
Some high school	1 (0.0)
Prefer not to answer	6 (0.4)
Skipped	55 (3.3)
**Employment status**	
Retired	770 (46.4)
Full time employment	502 (30.2)
Doing volunteer or unpaid work	181 (10.9)
Part time employment	160 (9.6)
Self-employed	152 (9.2)
Homemaker	96 (5.8)
Disabled/unable to work	36 (2.2)
Looking for paid work	16 (1.0)
Student	16 (1.0)
Prefer not to answer	11 (0.7)
Skipped	52 (3.1)
**Annual household income, $**	
<50,000	339 (20.4)
50,000–74,999	314 (18.9)
75,000–99,999	291 (17.5)
≥ 100,000	404 (24.3)
Don’t know/prefer not to answer	260 (15.7)
Skipped	53 (3.2)

### 3.2 Helpfulness of medications and supplements

Table 2 describes the results for participants’ beliefs about the levels of helpfulness of different medications and supplements for brain health. Across the selected medications and supplements, 46–64% of respondents reported not knowing or skipped the item regarding their helpfulness to improve brain health. Among the selected medications and supplements, fish oil was most frequently reported to be very/somewhat helpful for brain health (42.6% of respondents), followed by vitamin E (24.3%), statins (19.2%), ginkgo biloba (17.5%), and hormones (estrogen or testosterone) (15.1%). On post-hoc analysis, a greater proportion of women reported hormones to be very/somewhat helpful, and a greater proportion of men reported statins to be very/somewhat helpful (Supplementary Table 1). Moreover, a greater proportion of younger respondents reported ginkgo biloba and fish oil to be very/somewhat helpful, whereas a greater proportion of older respondents reported statins to be very/somewhat helpful (Supplementary Table 2).

**Table 2:** Beliefs about Helpfulness or Harmfulness of Medications and Supplements for Brain Health (n = 1661).

**Pharmacological Agent**	**Respondents, No. (%)**
Level of ***helpfulness*** for a person’s brain health	
***Vitamin E***	–
Very/somewhat useful	403/1661 (24.3)
Not at all useful	247/1661 (14.9)
Don’t know/Skipped	981/1661 (59.1)
***Ginkgo biloba***	–
Very/somewhat useful	291/1661 (17.5)
Not at all useful	370/1661 (22.3)
Don’t know/Skipped	955/1661 (57.5)
***Hormones such as estrogen or testosterone***	–
Very/somewhat useful	250/1661 (15.1)
Not at all useful	313/1661 (18.8)
Don’t know/Skipped	1048/1661 (63.1)
***Fish oil***	–
Very/somewhat useful	708/1661 (42.6)
Not at all useful	174/1661 (10.5)
Don’t know/Skipped	764/1661 (46.0)
***Statin for cholesterol like Zocor or Lipitor***	–
Very/somewhat useful	319/1661 (19.2)
Not at all useful	243/1661 (14.6)
Don’t know/Skipped	1068/1661 (64.3)
Level of ***harmfulness*** for a person’s brain health	
***Proton pump inhibitor (PPI) such as Prilosec***	–
Very/somewhat harmful	301/1661 (18.1)
Not at all harmful	72/1661 (4.3)
Don’t know/Skipped	1285/1661 (77.4)
***Over-the-counter sleep aid such as Benadryl***	–
Very/somewhat harmful	522/1661 (31.4)
Not at all harmful	94/1661 (5.7)
Don’t know/Skipped	1043/1661 (62.8)

### 3.3 Harmfulness of medications and supplements

Table 2 also describes the results for participants’ beliefs about the levels of harmfulness of two medications classes for brain health. Between the two medication classes (PPIs and anticholinergics used as sleep aids), 63–77% of respondents reported not knowing or skipped the item regarding their harmfulness to brain health. Nearly one-third (31.4%) of respondents thought OTC sleep aids (such as diphenhydramine) were very/somewhat harmful to brain health; 18.1% of respondents reported PPIs to be very/somewhat harmful to brain health. On post-hoc analysis, no remarkable differences were noted for the respondents’ beliefs about the harmfulness of PPIs and OTC sleep aids by sex or age categories.

## 4 Discussion

In this study, we administered a web-based survey to assess patients’ beliefs about the helpfulness or harmfulness of various medications and supplements on brain health, with the goal of identifying opportunities to improve communication strategies regarding pharmaceutical use for dementia prevention. Overall, survey respondents largely reported not knowing the potential benefits and harms of different medications and supplements for brain health, indicating pervasive lack of awareness about current evidence. In addition, one out of four respondents reported benefits of vitamin E and nearly half of respondents reported benefits of fish oil on brain health; neither benefit is supported by current evidence. This study uniquely contributes to the literature by providing some of the only data available on patient beliefs about the potential benefits and harms of different medications and supplements on brain health.

The most comprehensive report on the current evidence for the role of medications or supplements for cognitive protection was published in the 2017 National Academies’ landmark report, “*Preventing Cognitive Decline and Dementia: A Way Forward*” using results from two evidence-based reviews (Downey et al., 2017). Fink et al., 2018, Butler et al., 2018 each published a systematic review summarizing the evidence on efficacy and harms of pharmacological interventions and OTC supplements, respectively, to prevent or delay cognitive decline, mild cognitive impairment, or dementia. These reviews included trials of at least six months’ duration. Fink et al (2018) found that estrogen increased risk of dementia or a combined outcome of mild cognitive impairment or dementia based on results from one trial (low strength of evidence). In addition, statins did not alter dementia risk based on results from one trial (low to insufficient strength of evidence) (Fink et al., 2018). Despite this evidence, nearly 15% and 20% of survey respondents in our study reported belief that hormone and statin use, respectively, benefit brain health. It is important to note that some observational data has shown an association between statin use and reduced risk of dementia (Zissimopoulos et al., 2017), which may be why some respondents answered affirmatively. Moreover, it is likely that statins reduce long-term dementia risk through stroke risk reduction, but current clinical trials are of insufficient duration to answer this question directly.

Trials described by Butler et al (2018) showed no benefit in cognitive performance for vitamin E compared to placebo in women (moderate strength evidence); and no difference in dementia incidence for vitamin E compared to placebo in men (low strength evidence). Moreover, three trials demonstrated no protective benefits on Alzheimer’s dementia or cognitive performance for gingko biloba compared to placebo (low strength evidence) (Butler et al., 2018). Finally, seven trials showed no protective benefits on probable dementia incidence or cognitive performance for fish oil compared to placebo (low strength evidence) (Butler et al., 2018). Despite this evidence, approximately 25%, 18%, and 43% of survey respondents in our study reported belief that vitamin E, gingko biloba, and fish oil, respectively, benefit brain health.

The vast majority of the evidence base for potential harms of medications on brain health comes from epidemiological studies. Two of the most commonly studied medication classes assessed in contemporary dementia pharmacoepidemiology for their potential to increase dementia risk are PPIs and anticholinergics. Two studies from Germany (Gomm et al., 2016, Haenisch et al., 2015) and one from Taiwan (Tai et al., 2017) reported a higher risk of dementia in PPI users, whereas one US-based study reported no significant risk of dementia in long-term PPI users (Gray et al., 2018). These observational studies used different approaches to address confounding and measure PPI exposure and reported conflicting results (Song et al., 2019, Yang, 2018). While nearly 20% of survey respondents in our study reported some harm on brain health for PPI use, the vast majority (nearly 80%) reported not knowing.

Mounting evidence suggests that overall anticholinergic use is associated with increased dementia risk (Gray et al., 2015). More recent evidence, however, indicates that only certain anticholinergic sub-classes are associated with increased dementia risk (Coupland et al., 2019, Richardson et al., 2018). While nearly one-third of survey respondents in our study reported some harm on brain health for OTC sleep aid use, the majority (63%) reported not knowing. Future research is needed to elucidate the risk of long-term use of different anticholinergic sub-classes on dementia risk.

We did not assess the respondents’ source(s) of information specifically related to pharmaceutical use for brain health; however, future research – including both qualitative and quantitative methods – is needed on this important topic. Previous survey research has found that older adults’ preferred sources of health information are healthcare providers, friends and relatives, retirement community staff, newspapers, Internet, TV, and radio, with respondents trusting living sources (e.g., primary care physician) more than non-living sources (e.g., Internet) (Chauduhri, Le, White, Thompson, & Demiris, 2013). Additional qualitative research found that older adults routinely triangulate health information via multiple sources (Turner et al., 2018). Taken together, health communication on pharmaceutical effects on dementia risk is greatly needed, should prioritize healthcare provider-delivered information (e.g., via nurses or pharmacists), and must be routinely updated as new evidence emerges.

Health information-seeking behaviour is an active area of research, bolstered by efforts such as the Health Information National Trends Survey. The theoretical framework of this survey is based on the premise that there are two general stages in consumer-oriented health communication: 1) an awareness stage in which persuasive media push messages into awareness and contemplation, and 2) an information-seeking stage in which consumers pull information from sources (Nelson et al., 2004). Related to the topic of pharmaceutical effects on dementia risk, stage one is complicated by a variety of factors, including: media outlets at times sensationalizing research findings as well as over-the-counter pharmaceutical advertising (Huh et al., 2016). Stage two is complicated by potential information discrepancies encountered by patients when they seek information on pharmaceutical effects on dementia risk, leading to confusion and potentially behavior based on misinformation. A lot of confusion in this space stems from conflicting research findings (e.g., observational data of a protective effect of medication vs. interventional data of a neutral effect), among others. Moreover, product labelling is often insufficient to fully explain the complex and at times conflicting evidence on pharmaceutical effects and brain health. Healthcare providers need to understand the health communication ecosystem through which patients and their families navigate in order to provide effective dementia prevention communication.

There are important limitations to this study. We used a convenience sample from a single region in the US, limiting the generalizability of these results to other populations. Our sample was more likely to be females of an older age compared to the entire KPW membership. More specifically, among the more than 617,000 KPW members as of March 2018, 54.3% were female and 40.1% were 55 years of age and older compared to our sample where most respondents were older females. Future research on patient beliefs about the role of medications and supplements in dementia prevention is needed in more diverse and representative populations. We were also unable to calculate a survey response rate since we could not measure how many people opened the electronic newsletter. However, we viewed results as hypothesis-generating for future research efforts. In addition, survey respondents are likely different from those not responding, a source of possible selection bias. In order to keep the survey focused and brief, we prioritized key domains over clinical characteristics, such as medical history. Patients taking multiple chronic medications or supplements likely have different beliefs on this topic than those not taking medications. Future surveys should consider collecting such information. Finally, in order to minimize respondent burden, we did not include an exhaustive list of medications and supplements (e.g., B vitamins) that may support brain health. Notwithstanding these limitations, our study uniquely provides some of the only data available on patient beliefs about this fast-changing topic.

In conclusion, survey respondents were largely not aware of current evidence on the potential benefits and harms of different medications and supplements for brain health. In addition, respondents reported benefits of supplements on cognition that is not supported by current evidence. Improved health communication on pharmaceutical effects on dementia risk is greatly needed, and its development and dissemination should involve healthcare providers, patients, and media outlets working together.

## Funding

This work was supported by the Agency for Healthcare Research & Quality under Grant K12HS022982. The funding source had no involvement in the study design, collection, analysis, or interpretation of data, writing of the report, or the decision to submit the article for publication.

## Declaration of interest statement

The authors have no conflicts of interest to report.

## Data availability statement

Data are available from the corresponding author by request.

## Human participants protection

The Institutional Review Board of the Kaiser Permanente Washington Health Research Institute approved all procedures involving human subjects.

## CRediT authorship contribution statement

**Zachary A. Marcum:** Conceptualization, Data curation, Funding acquisition, Investigation, Methodology, Project administration, Supervision, Writing - original draft, Writing - review & editing. **Sarah D. Hohl:** Formal analysis, Writing - review & editing. **Douglas Barthold:** Writing - original draft, Writing - review & editing. **Oleg Zaslavsky:** Writing - original draft, Writing - review & editing. **Eric B. Larson:** Conceptualization, Funding acquisition, Supervision, Writing - original draft, Writing - review & editing. **Shelly L. Gray:** Conceptualization, Funding acquisition, Supervision, Writing - original draft, Writing - review & editing.
