# Supplementary material for: Beliefs about benefits and harms of medications and supplements for brain health
Source: Prev Med Rep. 2020 Jan 25;17:101060. doi: 10.1016/j.pmedr.2020.101060 (PMC6995253; doi:10.1016/j.pmedr.2020.101060)
Supplement: Supplementary data 1 [file mmc1.docx]

Supplementary Material

**Supplementary Figure 1**. Survey Instrument

**Dementia Prevention Survey**

For each of the following products, please mark if you think it is very useful, somewhat useful, or not at all useful for improving a person’s brain health:

Vitamin E

Very useful

Somewhat useful

Not at all useful

Don’t know

Ginkgo biloba

Very useful

Somewhat useful

Not at all useful

Don’t know

Hormones such as estrogen or testosterone

Very useful

Somewhat useful

Not at all useful

Don’t know

Fish oil

Very useful

Somewhat useful

Not at all useful

Don’t know

“Statin” for cholesterol like Zocor or Lipitor

Very useful

Somewhat useful

Not at all useful

Don’t know

How harmful do you think the following medications are for a person’s brain health?

Proton pump inhibitor (PPI) such as Prilosec

Very harmful

Somewhat harmful

Not at all harmful

Don’t know

Over-the-counter sleep aid such as Benadryl

Very harmful

Somewhat harmful

Not at all harmful

Don’t know

What is your age?

20 or younger

21-30

31-40

41-50

51-60

61-70

71-80

81-90

91 or older

What is your gender identity?

Male

Female

Non-binary

Other, Specify: _____________________________

Prefer not to answer

What was the highest level of school you completed?

Up to 8^th^ grade

Some high school

High School graduate or GED

Technical Trade or vocational school after high school

Some college (including an associates degree)

College or university graduate (4-year degree)

Some Post graduate or professional school after college

Post graduate or professional degree

Don’t know

Prefer not to answer

Which of the following best describes your current employment status? *Please choose all that apply*.

Employed full time

Employed part time

Retired

Self-employed

Doing volunteer or unpaid work

Looking for paid work

Disabled/Unable to work

Homemaker

Student

Prefer not to answer

What is your approximate annual household income before tax?

Less than $15,000

$15,000 to less than $25,000

$25,000 to less than $35,000

$35,000 to less than $50,000

$50,000 to less than $75,000

$75,000 to less than $100,000

$100,000 or more

Don’t know

Prefer not to answer

Are you of Hispanic or Latino/a origin or descent?

Yes,

No,

Don’t know

Prefer not to answer

What best describes your race? *Please choose all that apply*.

American Indian or Alaska Native

Asian

Black or African American

Native Hawaiian or Other Pacific Islander

White

Prefer not to answer

Other, please specify.

**Supplementary Table 1**. Beliefs about Helpfulness or Harmfulness of Medications and Supplements for Brain Health Results by Sex

| **Pharmacological Agent** | | |
| --- | --- | --- |
| Level of ***helpfulness*** for a person’s brain health | | |
|  | **Male (%)** | **Female (%)** |
| ***Vitamin E***  Very/somewhat useful  Not at all useful  Don’t know/Skipped  ***Ginkgo biloba***  Very/somewhat useful  Not at all useful  Don’t know/Skipped  ***Hormones such as estrogen or testosterone***  Very/somewhat useful  Not at all useful  Don’t know/Skipped  ***Fish oil***  Very/somewhat useful  Not at all useful  Don’t know/Skipped  ***Statin for cholesterol like Zocor or Lipitor***  Very/somewhat useful  Not at all useful  Don’t know/Skipped | --  27.7  17.0  55.3  --  14.5  26.3  59.2  --  9.5  26.0  64.5  --  41.4  12.8  45.8  --  25.2  13.2  61.6 | --  24.7  15.3  60.0  --  19.2  22.8  58.0  --  17.1  18.5  64.4  --  44.6  10.4  45.0  --  18.6  15.5  65.9 |
| Level of ***harmfulness*** for a person’s brain health | | |
|  | **Male (%)** | **Female (%)** |
| ***Proton pump inhibitor (PPI) such as Prilosec***  Very/somewhat harmful  Not at all harmful  Don’t know/Skipped  ***Over-the-counter sleep aid such as Benadryl***  Very/somewhat harmful  Not at all harmful  Don’t know/Skipped | --  18.4  5.4  76.2  --  32.2  6.3  61.5 | --  18.2  4.3  77.5  --  31.6  5.7  62.7 |

**Supplementary Table 2**. Beliefs about Helpfulness or Harmfulness of Medications and Supplements for Brain Health Results by Age Category

| **Pharmacological Agent** | | | |
| --- | --- | --- | --- |
| Level of ***helpfulness*** for a person’s brain health | | | |
|  | **18-50 yrs (%)** | **51-70 yrs (%)** | **71+ yrs^*^ (%)** |
| ***Vitamin E***  Very/somewhat useful  Not at all useful  Don’t know/Skipped  ***Ginkgo biloba***  Very/somewhat useful  Not at all useful  Don’t know/Skipped  ***Hormones such as estrogen or testosterone***  Very/somewhat useful  Not at all useful  Don’t know/Skipped  ***Fish oil***  Very/somewhat useful  Not at all useful  Don’t know/Skipped  ***Statin for cholesterol like Zocor or Lipitor***  Very/somewhat useful  Not at all useful  Don’t know/Skipped | --  27.9  9.0  63.1  --  26.8  14.7  58.5  --  13.4  16.5  70.1  --  52.4  6.5  41.1  --  10.0  13.0  77.0 | --  24.1  16.4  59.5  --  18.7  23.3  58.0  --  17.1  19.8  63.1  --  44.3  10.8  44.9  --  20.4  16.1  63.5 | --  26.6  17.6  55.8  --  11.9  29.8  58.3  --  14.3  22.8  62.9  --  37.7  14.0  48.3  --  25.5  14.5  59.9 |
| Level of ***harmfulness*** for a person’s brain health | | |  |
|  | **18-50 yrs (%)** | **51-70 yrs (%)** | **71+ yrs (%)** |
| ***Proton pump inhibitor (PPI) such as Prilosec***  Very/somewhat harmful  Not at all harmful  Don’t know/Skipped  ***Over-the-counter sleep aid such as Benadryl***  Very/somewhat harmful  Not at all harmful  Don’t know/Skipped | --  14.6  6.0  79.4  --  29.9  9.4  60.7 | --  20.4  4.2  75.4  --  32.7  5.6  61.7 | --  15.7  4.3  80.0  --  31.9  4.0  64.1 |

^*^Age categories 71-80 and >81 combined due to sample size
